# Survival of Toddler with Aortoesophageal Fistula after Button Battery Ingestion

**DOI:** 10.1155/2021/5557054

**Published:** 2021-10-05

**Authors:** Hannah Gibbs, Rishabh Sethia, Patrick I. McConnell, Jennifer H. Aldrink, Toshiharu Shinoka, Kent Williams, Kris R. Jatana

**Affiliations:** ^1^College of Medicine, The Ohio State University, Columbus, OH, USA; ^2^Department of Otolaryngology–Head & Neck Surgery, The Ohio State University Wexner Medical Center, Columbus, OH, USA; ^3^Department of Cardiothoracic Surgery, Nationwide Children's Hospital, Columbus, OH, USA; ^4^Division of Pediatric Surgery, Department of Surgery, Nationwide Children's Hospital, The Ohio State University College of Medicine, Columbus, OH, USA; ^5^Division of Pediatric Gastroenterology, Nationwide Children's Hospital, Columbus, OH, USA; ^6^Department of Pediatric Otolaryngology, Nationwide Children's Hospital, Columbus, OH, USA

## Abstract

Button batteries (BBs) are found in many households and are a source of esophageal foreign body in the pediatric population. Upon ingestion, significant caustic injury can occur within 2 hours leading to tissue damage and severe, potentially fatal sequelae. Aortoesophageal fistula (AEF) is a rare complication that nearly always results in mortality. We report a rare case of a toddler who developed an AEF after BB ingestion and survived following staged aortic repair. There should be a high index of suspicion for this complication with the history of BB ingestion and presence of hematemesis, hemoptysis, or melena.

## 1. Introduction

**Figure 1:**
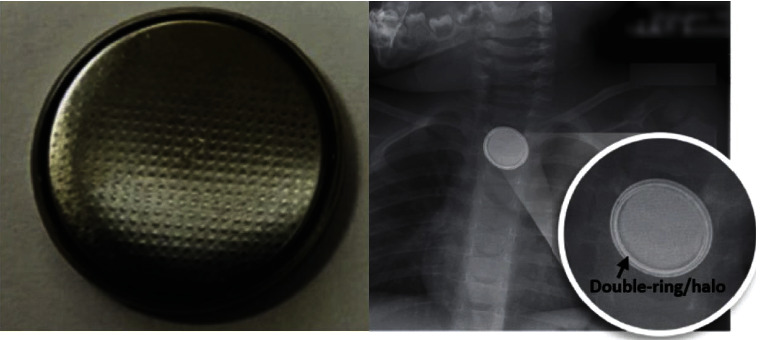
Button batteries are metallic, disc-shaped objects (left). They may be recognized on radiographs by identifying the characteristic “double-ring” or “halo” sign (right), reproduced with permission of KR Jatana.

Children under 5 years of age in the United States ingest nearly 70,000 foreign bodies annually, of which nearly 3,000 are button battery (BB) ingestions [1, 2]. BBs are disc-shaped metallic objects found in many household objects (Figure 1); the most common sources of ingested BBs include remote controls, games and toys, hearing aids, watches, calculators, and flashlights [3]. The incidence of BB ingestions and associated morbidity and mortality increased in the past two decades, especially with the introduction of more technologically advanced toys and electronics in the household [4]. One study reported that major injuries such as esophageal perforation or stricture, tracheoesophageal fistula, vocal cord paralysis, spondylodiscitis, or fistulization into major vessels occurred in 12.6% of children <6 years old [2].

BBs are easily ingested by children, and this event is not always witnessed. Following ingestion, patients may be initially asymptomatic or present with vague, nonspecific symptoms similar to common viral illnesses. BBs also have a uniquely ideal size and shape to become lodged in the pediatric esophagus. This is extremely worrisome as severe esophageal injury and liquefactive necrosis can occur within two hours of ingestion due to an ensuing alkaline reaction [5]. Furthermore, if not neutralized after removal, a persistent alkaline environment can continue to cause tissue damage to the surrounding tissues postremoval [2].

Due to its proximity to the trachea, the aorta, and other major vessels, BBs lodged in the esophagus can cause potentially life-threatening injuries. To date in 2021, 67 deaths caused by BBs have been reported and most of these fatalities involved BBs impacted in the esophagus [6]. Aortoesophageal fistulas (AEF) and fistulization into other major vessels are the most acute life-threatening outcome of esophageal BB impaction, as these injuries are nearly always fatal secondary to massive exsanguination. In cases where active bleeding ceases (sentinel bleed) is when there is a chance for survival when the patient has enough blood volume reserve and/or resuscitation measures immediately available. In this study, we present a rare case of a toddler who presented with AEF sentinel bleed after BB ingestion and survived after successful staged aortic repair.

## 2. Case Report

**Figure 2:**
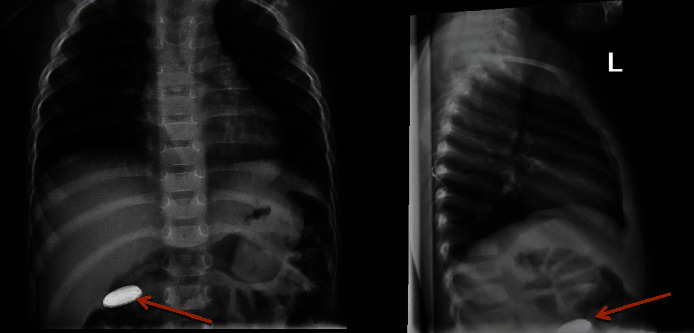
CXR with PA (left) and lateral (right) views of the rounded radiopaque foreign body with double ring appearance peripherally concerning for button battery in the right upper quadrant at the expected region of the duodenum. There is typically approximately 10% magnification on measurement of metallic foreign bodies, consistent with a 20 mm 3V lithium BB.

**Figure 3:**
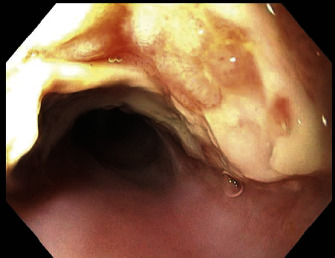
EGD revealing grade 2A partially circumferential caustic injury with thickening of mucosa, exudates, and ulcerations in the upper third of the esophagus without active bleeding.

**Figure 4:**
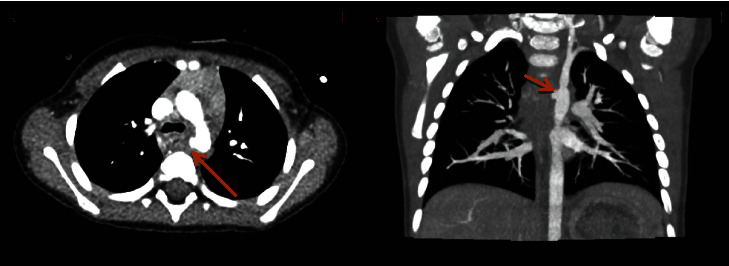
Presenting with hematemesis 7 days after initial EGD, CT angiogram axial (left) and coronal (right) views revealing pseudoaneurysm along the posteromedial wall near the distal aortic arch/proximal descending thoracic aorta just caudal to the takeoff of the left subclavian artery with no evidence of active bleeding.

A 16-month-old girl presented to the emergency department (ED) with a 2-week history of intermittent nausea and vomiting, as well as cough, fevers, and anorexia. Exam was unremarkable. A CXR was obtained which revealed a suspected BB in the right upper quadrant (Figure 2). She was taken to the operating room (OR) and underwent EGD which revealed a 5 cm long, grade 2A, anterior midesophageal caustic injury with no evidence of bleeding (Figure 3). The BB was beyond the duodenum and was beyond the reach of endoscopic removal. Seven days after initial EGD, the patient developed hematemesis and was found to have hemoglobin of 7.1 g/dL. A CT angiogram (CTA) was obtained which revealed a pseudoaneurysm at the distal aortic arch beyond the left subclavian artery (Figure 4). She went emergently to the OR and from a median sternotomy and using cardiopulmonary bypass but avoiding cardiac cardioplegic arrest, she underwent an intraaortic patch repair (CardioCel^®^, LeMaitre Vascular Inc., Burlington, MA) via the transverse aortic arch. The pericardium was irrigated, but due to lack of adequate access to the posterior mediastinum, the AEF was not taken down at that time, instead opting for a staged repair. She was continued on broad-spectrum IV antibiotics for mediastinitis prophylaxis and kept NPO. She was also found to have paralysis of the left vocal cord which appeared to be in paramedian position on flexible fiberoptic laryngoscopy.

**Figure 5:**
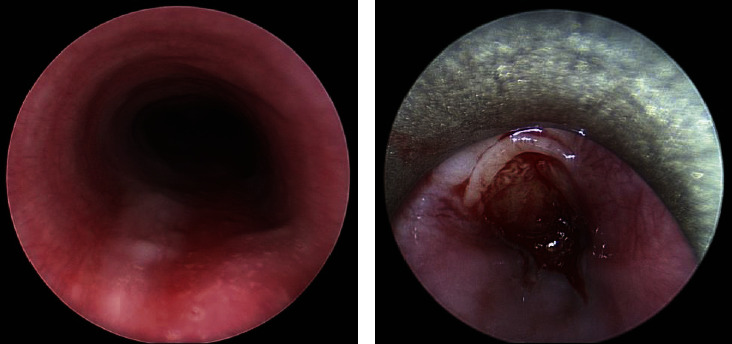
Second surgery in the staged repair of the AEF after the first surgery controlled the active aortic hemorrhage. Rigid bronchoscopy (left) showing no evidence of tracheoesophageal fistula. Rigid esophagoscopy (right) revealing the anterior esophageal injury near start of the midesophagus with friable, healing granulation tissue consistent with a likely negative pole of prior esophageal button battery facing anteriorly.

**Figure 6:**
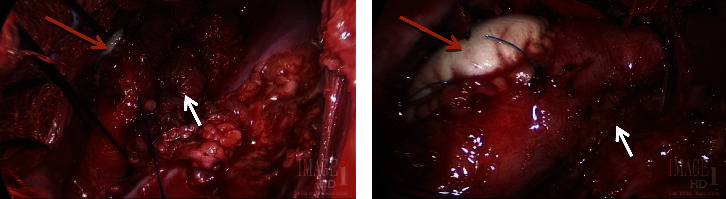
Intraoperative images of intercostal muscle flap (white arrow) second stage surgical repair of aortoesophageal fistula. Acellular collagen bioscaffold (CardioCel®, LeMaitre Vascular Inc., Burlington, MA) was also used (orange arrow).

**Figure 7:**
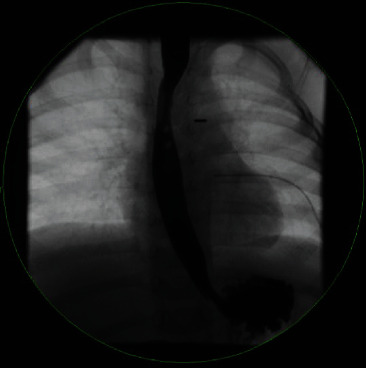
Esophagram obtained 10 days following repair showing no evidence of leak.

Four days later, she underwent a planned staged separation of the AEF. Panendoscopy at that time demonstrated granulation tissue with some active bleeding within the anterior esophagus corresponding with the negative pole of the prior esophageal BB facing anteriorly (Figure 5). Through a left posterior thoracotomy incision, an intercostal muscle flap was created, the esophageal fistula was opened, devitalized esophagus was debrided, and the intercostal muscle flap was interposed (Figure 6) and sutured circumferentially to the esophagostomy, repairing the esophagus. The external aortic wall was not directly repaired or patched. A gastrostomy tube was placed surgically for ongoing enteral nutrition. Postoperatively, her course was uneventful. She was started on postpyloric tube feeds and underwent an esophagram 10 days postrepair which showed no evidence of leak (Figure 7). Her feeds were changed to intragastric feeds, and she underwent a video swallow study prior to discharge allowing her diet to be advanced to a regular diet. She was discharged 28 days after admission.

**Figure 8:**
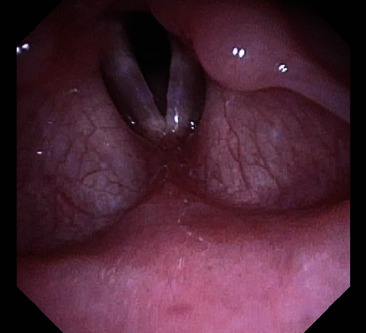
Flexible fiberoptic laryngoscopy 2.5 months after open chest repair reveals improving left vocal cord paresis.

Subsequent esophagrams at 4 weeks, 6 weeks, 5 months, and 7 months postrepair demonstrated no stricture formation. Upon repeat flexible laryngoscopy 10 weeks after her repair, vocal cord mobility appeared to be improved with demonstration of left vocal cord paresis rather than complete paralysis (Figure 8). Her vocal cord paresis appeared to be nearly resolved 17 months after repair and she had no swallowing, voice, nor breathing concerns. Nearly 18 months after her surgical repair, she continues to do well, and her gastrostomy tube has been removed.

## 3. Discussion

BBs are uniquely dangerous pediatric foreign bodies that are present in many common household items. Upon ingestion, BBs create a caustic alkaline environment and can induce tissue damage within two hours that can continue to progress even after removal [2, 5]. Given their ideal shape and size, BBs can become lodged in the pediatric esophagus prompting emergent identification and removal to prevent serious complications such as esophageal perforation or stricture, tracheoesophageal fistula, vocal cord paralysis, spondylodiscitis, and major vessel damage [2]. Although patients may be asymptomatic or present with nonspecific symptoms initially, identification with early radiographic imaging followed by emergent removal is the standard of care for a majority of BB ingestions [2, 7]. Certain mitigation strategies have been recommended to prevent progression of tissue damage resulting from alkaline-induced liquefactive tissue necrosis. In a household setting, if a BB ingestion is suspected, the child should be taken to the emergency department immediately [7, 8]. Recent review studies have described the management of esophageal BBs including the use of mitigation strategies to slow initial or progression of injury, such as preremoval (honey or sucralfate) and postremoval (0.25% acetic acid), respectively [9–12].

According to the National Capital Poison Center, 67 fatalities and 254 nonfatal severe esophageal or airway injuries have been reported following BB ingestion as of 2021 [6]. Based on an internal review of the literature, 48 cases of BB ingestion leading to esophageal-vascular fistula were identified. Of these cases, 41 resulted in death mainly due to exsanguination and only 7 survived. We present the 8^th^ reported survival of a child affected by such an injury. For the cases resulting in death with known reported information, the average age was 2.16 years, there were 25 females and 13 males, and the most common symptom present was hematemesis (26/41, 63%). Less common symptoms included vomiting, fever, abdominal pain, coughing, melena, dysphagia, throat pain, anorexia, and epistaxis. In many cases, the length of exposure in these cases resulting in death was unknown. The details for the cases of survival are given in Table 1. For patients who survived, the average age was 2.33 years, most were female, and ingested either lithium batteries or unknown type. All patients who survived presented with hematemesis.

Since mortality from these injuries is high, attention should be given to the management of the few cases in which survival did occur. The child in case #1 presented 3 weeks after BB removal with hematemesis and underwent excision of the fistula, end-to-end anastomosis, and patching with pericardium [13]. Case #2 reports surgical repair of the AEF without further details, and the patient was noted to develop bilateral lower extremity paralysis [14]. In case #3, bleeding was so severe that extensive blood and clots in the esophagus initially prevented identification of the primary bleeding site. The patient experienced massive hematemesis and severe hemodynamic shock, requiring multiple blood transfusions and vasopressors. An endoscopic dilation balloon was inflated in the midesophagus over the bleeding site once identified, and then, a stent was placed over the ruptured thoracic aorta and dilated. Once hemodynamically stable, hemostatic endoscopic powder (Hemospray) was sprayed on bleeding sites [15]. Case #4 reports that the child presented one month after BB removal with massive hematemesis, and the aortic fistula was closed using 2 layers of 5–0 Prolene sutures. This child subsequently underwent reconstructive surgery 6 months later [16]. In case #5, the patient presented with recurrent hematemesis 10 days and 17 days after BB removal and underwent excision of AEF with anastomosis and coverage with xenopericardium [17]. In case #6, the toddler experienced massive bleeding and two episodes of cardiac arrest. The exact mechanism of repair is not well documented but involved a type of flap and the use of a hypothermia protocol and ECMO [14]. After BB removal, the child in case #7 had undergone serial MRI exams that showed improvement before she was discharged, but she returned to the hospital with hematemesis 25 days after BB removal. Outpouching and intimal injury of the descending aorta was managed with placement of a 12 mm × 34 mm 8 zig premounted covered Cheatham Platinum (CP) stent. It was specifically noted that acetic acid had not been used to irrigate the area of esophageal injury after initial BB removal. Furthermore, despite undergoing serial imaging after removal that suggested improvement, she still eventually developed an aortoesophageal fistula [18]. In our case (#8), the aortic opening was closed with the acellular bovine pericardium followed by fistula takedown and intercostal muscle flap repair of the esophagus.

In our case, we highlight the similarities to previously reported data which suggest a high prevalence of unwitnessed BB ingestion; nearly 56.2% of major outcome cases pertaining to BBs were unwitnessed (only 30.1% were witnessed) in one study by Litovitz et al. [2]. Also, as in many other cases, our patient initially presented with vague, nonspecific symptoms leading to potentially delayed identification, and a prolonged exposure period following BB ingestion. The commonality of vague symptoms following unwitnessed BB ingestions in fatal and nonfatal severe injury cases suggests that clinicians must consider strongly and if concerned, further evaluate for BB ingestion to avoid catastrophic consequences. Finally, it is important to highlight that our patient developed hematemesis and was found to have AEF 7 days after initial EGD and for an unknown time period after unwitnessed BB ingestion. This correlates to the literature which suggests delayed presentation of vascular fistulas ranging from days to weeks following BB ingestion and removal [3]. This is likely related to the persistent alkaline-induced liquefactive necrosis following removal and may be minimized by use of the 0.25% acetic acid irrigation [8]. Even when a BB is not in the esophagus, 0.25% acetic acid irrigation of esophageal injury could still be considered to neutralize the high pH in an attempt to slow injury progression. A specific published case that highlights the importance of neutralizing tissue after BB removal involves a 15-month-old boy who ingested two 3V lithium BBs, creating a severe circumferential esophageal injury. Although the grade of esophageal injury was not reported, there was necrosis noted, making this at least a grade 3A injury. After endoscopic removal 8 hours postingestion and neutralization with 100 mL of 0.25%, his clinical outcome was better than initially expected and he recovered without stricture formation [19]. In both fatal and nonfatal vascular fistulas, hematemesis is a key presenting symptom. If present, providers must thoroughly investigate as to not overlook a possible AEF.

Given the prolonged time for potential development of serious complications, clinicians must not only be vigilant upon initial evaluation of pediatric patients with concern for BB ingestion but these patients must also be monitored for delayed complications following removal. Certainly, any unexplained signs or symptoms in a patient with history of BB ingestion should warrant further investigation. Although clinicians should maintain a high index of suspicion for serious complications in all BB ingestion patients, certain predictive features such as age ≤5 years of age, prolonged time of impaction, anterior anatomic directionality of the BB negative pole, and specific higher-risk BB parameters such as diameter >20 mm and 3 volts may be helpful to guide management and surveillance [10, 20]. There are limited data about the role of imaging, and it is not yet known what the optimal timing, modality, and parameters to assess are. Thus, some guidelines about imaging are considerations that may be taken. If there is any concern for vascular fistula, such as with development of hematemesis, cross-sectional imaging with chest CTA or MRI with contrast should be obtained without delay. In cases of midesophageal BB removal with severe mucosal injury, screening with chest CTA could be considered. Only 13% of patients who ingested BBs between 2008 and 2017 received contrast CT or MRI imaging, but in recent years, the use of CTA or MRI in BB ingestion cases has greatly increased [21]. The North American Society for Pediatric Gastroenterology, Hepatology, and Nutrition (NASPGHN) suggests considering (but not mandating) CTA or MRI to exclude aortic injury or to determine proximity to aorta if there is evidence of esophageal injury. As far as monitoring, NASPGHN also suggests serial MRI every 5–7 days until the injury observably moves away from the aorta [22]. Early involvement of cardiothoracic surgery and other appropriate ECMO teams is also critical as a multidisciplinary approach is required to manage this life-threatening complication. In a review of BB ingestion management, one institution proposed using a specialized cardiac OR for higher-risk cases, whereas lower-risk cases could be performed in the general OR [23].

To help reduce BB-related injuries, the National Button Battery Task Force (BBTF) is formed in 2012 and employed the following mission statement.

“A collaborative effort of representatives from relevant organizations in industry, medicine, public health, and government to develop, coordinate, and implement strategies to reduce the incidence of button battery injuries in children.”

The National BBTF works to offer comprehensive guidance pertaining to BB research, data collection and analysis, management guideline and algorithm creation, advocacy, and education [24].

It is also important to note that the incidence of BB injuries is underreported. Over 400+ pediatric specialists who managed over 32,000 foreign injuries like BB were surveyed, and it was found that only 11% of BB injuries and 4% of overall foreign body injury cases were reported to a data source. About 92% of respondents stated they would contribute to injury statistics if it were more convenient. The Global Injury Research Collaborative (GIRC, http://www.globalirc.org), a nonprofit, produced a smartphone application (iOS and Android) to address this issue. The free “GIRC App” provides a convenient, user-friendly method to report foreign body injuries anonymously. This deidentified, HIPAA-compliant information provides data that will help stratify and mitigate foreign body injury cases [24]. Physicians involved in the management of these injury cases should report them to help collect the data needed to prevent future injuries in children.

## 4. Conclusion

BB ingestion is unfortunately often unwitnessed and can be life-threatening if not quickly recognized and treated. Diagnosis may be difficult due to asymptomatic presentation or vague symptoms. Prompt identification and endoscopic removal are keys to prevent serious complications. AEF is an often fatal consequence of BB ingestion which can present with hematemesis days to weeks after BB removal. There are limited cases of patients who have survived this complication. To prevent death, clinicians must maintain a high index of suspicion for AEF and other serious sequelae in patients with a history of BB ingestion.

## Figures and Tables

**Table 1:** Known survival cases of children with aortoesophageal fistula.

Case	Study	Age (years)	Gender	BB type	Length of BB exposure (hours)	Presenting symptoms	Sentinel bleed	Outcome and associated complications
1	Spiers, 2012	0.83	M	Unknown	14	Hematemesis	Yes	Esophageal stricture requiring continued intermittent balloon dilations

2	NCPC, 2017	2	F	Lithium	Unknown	Hematemesis	Unknown	Gastrostomy tube; paralysis of the legs

3	Granata, 2018	3	F	Lithium (CR 2025)	Unknown	Abdominal pain and hematemesis	No, but reduction in hemorrhage with 20 mm balloon in the esophagus, rapid shock resuscitation, and prompt intervention	Nasoduodenal tube for one month

4	Mahajan, 2018	3	F	Unknown	Unknown	Hematemesis	Yes	Gastrostomy-jejunostomy tube; reconstructive surgery 6 months postop

5	Bartkevics, 2019	1	F	Lithium (20 mm)	Unknown	Hematemesis, melena	Yes	No associated complications reported

6	NCPC, 2020	1.5	F	Lithium (20 mm)	Unknown	Hematemesis	Unknown	Gastrostomy tube

7	Sinclair, 2021	6	F	Unknown (21 mm)	6	Hematemesis	Yes	Temporary nasogastric tube

8	Current case, Gibbs, 2021	1.33	F	Lithium (20 mm, 3V)	Unknown	Hematemesis	Yes	Temporary gastrostomy tube (now removed); left vocal cord paresis (improving); no esophageal stricture

## Data Availability

The used to support this study are available at the National Capital Poison Center at https://www.poison.org/battery and at PubMed at https://pubmed.ncbi.nlm.nih.gov/. These prior studies (and datasets) are cited at relevant places within the text as references [1–24].
